# Maintenance Cognitive Stimulation Therapy (CST) for dementia: A single-blind, multi-centre, randomized controlled trial of Maintenance CST vs. CST for dementia

**DOI:** 10.1186/1745-6215-11-46

**Published:** 2010-04-28

**Authors:** Elisa Aguirre, Aimee Spector, Juanita Hoe, Ian T Russell, Martin Knapp, Robert T Woods, Martin Orrell

**Affiliations:** 1 Department of Mental Health Sciences, University College London, Charles Bell House, 67-73 Riding House Street, London, UK; 2 Department of Clinical, Educational and Health Psychology, University College London, 1-19 Torrington Place, London, UK; 3 North Wales Organisation for Randomised Trials in Health and Social Care, Institute of Medical and Social Care Research, Bangor University, Ardudwy Hall, Normal Site, Holyhead Road, Bangor, UK; 4 Centre for Economics of Mental Health, Institute of Psychiatry, London, UK; 5 Dementia Services Development Centre Wales, Institute of Medical and Social Care Research, Bangor University, Ardudwy Hall, Normal Site, Holyhead Road, Bangor, UK

## Abstract

**Background:**

Psychological treatments for dementia are widely used in the UK and internationally, but only rarely have they been standardised, adequately evaluated or systematically implemented. There is increasing recognition that psychosocial interventions may have similar levels of effectiveness to medication, and both can be used in combination. Cognitive Stimulation Therapy (CST) is a 7-week cognitive-based approach for dementia that has been shown to be beneficial for cognition and quality of life and is cost-effective, but there is less conclusive evidence for the effects of CST over an extended period.

**Methods/Design:**

This multi-centre, pragmatic randomised controlled trial (RCT) to assess the effectiveness and cost-effectiveness of Maintenance CST groups for dementia compares a intervention group who receive CST for 7 weeks followed by the Maintenance CST programme once a week for 24 weeks with the control group who receive CST for 7 weeks, followed by treatment as usual for 24 weeks.

The primary outcome measures are quality of life of people with dementia assessed by the QoL-AD and cognition assessed by the ADAS-Cog. Secondary outcomes include the person with dementia's mood, behaviour, activities of daily living, ability to communicate and costs; as well as caregiver health-related quality of life. Using a 5% significance level, comparison of 230 participants will yield 80% power to detect a standardised difference of 0.39 on the ADAS-Cog between the groups. The trial includes a cost-effectiveness analysis from a public sector perspective.

**Discussion:**

A pilot study of longer-term Maintenance CST, offering 16 weekly sessions of maintenance following the initial CST programme, previously found a significant improvement in cognitive function (MMSE) for those on the intervention group. The study identified the need for a large-scale, multi-centre RCT to define the potential longer-term benefits of continuing the therapy. This study aims to provide definitive evidence of the potential efficacy of maintenance CST and establish how far the long-term benefits can be compared with antidementia drugs such as cholinesterase inhibitors.

**Trial Registration:**

ISRCTN26286067

## Background

Psychological treatments for dementia such as Reality Orientation (RO) and Reminiscence have been in use for nearly half a century, and are widely used in the UK and internationally, but often have not been standardised, adequately evaluated or systematically implemented. A number of systematic reviews of psychosocial interventions are now available [1-3], as well as a number of Cochrane reviews of specific approaches [e.g. [4,5]]. The Spector review of RO [5] was used to develop a seven-week evidence-based Cognitive Stimulation Therapy (CST) programme for people with dementia [6]. A total of 201 people with dementia were recruited for this single-blind, multi-centre RCT from 23 day centres and residential homes in greater London. The CST group improved significantly on the main outcome measures (cognition and quality of life). CST compared favourably with cholinesterase inhibitors for Alzheimer's disease in terms of numbers needed to treat (NNT) [6] and the economic analysis showed that CST was likely to be cost-effective [7]. The recent UK guidelines on dementia [8] recommend that all people with mild/moderate dementia should be 'given the opportunity to participate in a structured group cognitive stimulation programme'.

The evidence for the long-term effects of CST is less conclusive. Feedback from the CST training and previous studies [6] suggested that service users preferred a longer-term programme after the seven-week CST intervention. A number of studies have looked at the longer-term effects of cognitive stimulation and related interventions. A recent pilot study of longer-term CST (maintenance CST) [9], offering 16 weekly sessions of maintenance, following the initial CST programme, found a significant improvement in cognitive function (MMSE) for those receiving ongoing Maintenance CST. This lasted nearly 6 months in comparison to a group of CST only and controls (p = 0.012). The study identified the need for a large-scale, multi-centre RCT to define the potential longer-term benefits of MCST for dementia. A number of other studies have looked at the longer-term effects of cognitive stimulation and related interventions in combination with cholinesterase inhibitors. Two recent RCTs found that over 6 months, cognitive stimulation and cholinesterase inhibitors in combination, were more effective than cholinesterase inhibitors alone [10,11]. There is a distinct overlap between what is described as Reality Orientation (RO) and Cognitive Stimulation. Both programmes often describe similar features, whilst more emphasis is placed on re-learning orientation information in RO, Cognitive Stimulation focuses on implicit information processing. Studies have suggested that with a RO intervention, the cognitive benefits gained were lost after 10 weeks [12] and one month [13] after stopping the programme. However, Wallis et al. [13] found that behavioural functioning continued to improve 10 weeks after the RO programme terminated. Other studies have suggested that longer programmes of RO may have longer-term effects [14,15]. Zanetti et al. [14], found, in a RCT, that the effect of RO on cognitive performance appeared to counteract the decline, observed in the control group, of 2.58 points on the MMSE. Similarly Metitieri et al. [15] found that people receiving long-term treatment declined in cognitive function significantly later, and remained at home longer than those receiving a shorter programme of RO. Both studies concluded that providing a longer term RO intervention was effective in slowing, at least temporarily, the dementia process. A novel aspect of the Zanetti et al study [14] was that they described an expected yearly decrease in Mini-Mental-State-Examination score [16] of 1.8-4.2 points in people with dementia. Therefore, it might be that pre-post comparisons in the studies which had used longer interventions (20 and 21 weeks) would have shown weaker results [17,18] as a result of the expected yearly decrease.

It is unclear how far maintenance programmes of CST might continue to benefit the participants. The studies included in the recent CST Cochrane review [19] ranged from using programmes of four weeks [20] to one year [21]. However, no relationship between the duration of the intervention and the outcome was shown. The trials with the strongest results and higher weight in the metanalyses [6,11] had 7 to 25 weeks of intervention respectively. Stronger evidence of their effectiveness is needed to support the long-term implementation of CST. It is also necessary to examine whether the combination of Maintenance CST with cholinesterase inhibitors for Alzheimer's disease is cost-effective and brings extra long-term benefits to cognition and quality of life of people with dementia. This paper describes the study protocol for a pragmatic RCT of CST versus CST followed by a 24 week maintenance CST programme undertaken with people experiencing mild to moderate dementia. This research programme aims to provide essential evidence to clarify the role of long-term CST interventions alone and in combination with cholinesterase inhibitors and the analyses of its cost-effectiveness.

## Methods/Design

**Figure 1:**
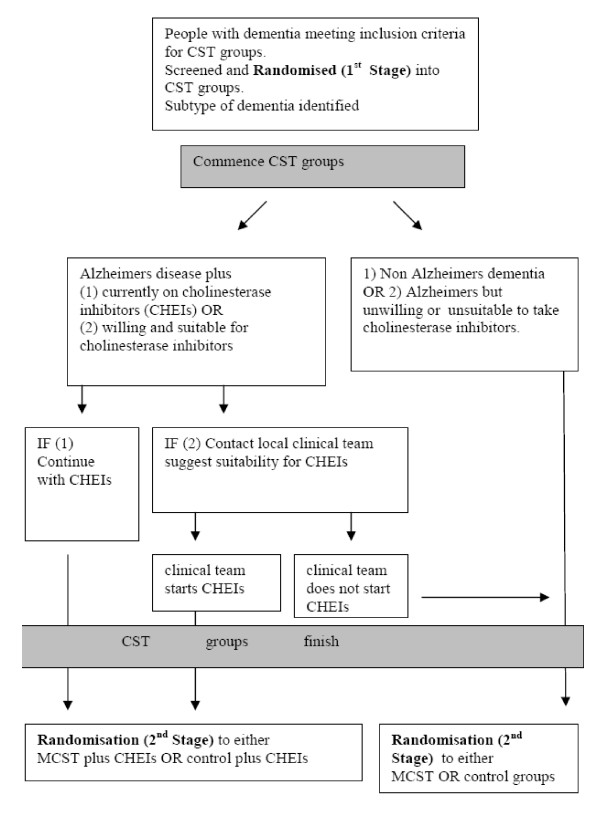
**Flow diagram of the trial; Trial and randomisation stages**.

The design is a single-blind, multi-centre, randomized controlled trial of Cognitive Stimulation Therapy (CST) groups for dementia vs. Maintenance CST groups (extra 24 sessions once a week) (Figure 1). After completion of the initial CST programme (twice weekly, 45-minute sessions for 7 weeks), participants are randomly allocated into treatment group (maintenance sessions once a week for 24 weeks) or control group (treatment as usual for 24 weeks).

People with Alzheimers disease are offered cholinesterase inhibitors by their local clinical team provided that they are willing and eligible (according to NICE guidelines [8]) for the medication. People currently on cholinesterase inhibitors will continue taking them. Participants enter the study after giving signed informed consent in accordance with the provisions of the Mental Capacity Act 2005. Ethical approval was obtained through the Multi-centre Research Ethics Committee (ref no. 08/H0702/68). The clinical trial is registered - ISRCTN 26286067.

### Participants

Recruitment to this trial is currently taking place through day centres, residential homes and Community Mental Health Teams (CMHTs) in the participating study centres, with at least a minimum of 14 potential participants. Half of the sample to be recruited from the community (50%) and the other half of the sample (50%) from care homes. Potential centres (day centres, CMHTs and residential homes) are screened for eligibility to determine whether there are sufficient numbers of potential participants with dementia, using the inclusion criteria flow chart. (Appendix 1). People with dementia meeting the inclusion criteria are recruited into the two CST groups that are running per centre (7 to 10 per group).

### Randomisation

The randomisation process in this trial is undertaken in two stages, randomisation 1 and randomisation 2. Figure 1 sets out the two-stage randomisation process. The allocation ratio at randomisation 1 stage is 1:1; into either group A or group B, with both groups receiving 7 weeks of CST. The allocation ratio at randomisation 2 stage is 1:1; into either the control group or treatment group. The sample is stratified to ensure that equal numbers of participants taking cholinesterase inhibitors are randomised into either the Maintenance CST or the control group. The North Wales Organisation for Randomised Trials in Health (NWORTH) is responsible for undertaking the remote randomisation. NWORTH is accredited as a Clinical Trials Unit by the UK Clinical Research Collaboration (UKCRC) and funded as part of the Clinical Research Collaboration Cymru, notably for HTA trials.

### Blinding

Although participants cannot be blinded to their allocated treatment, all follow-up data is gathered by interviewers blind to groups. However our experience in the CST trial, as shared by similar projects, is that participants may occasionally and inadvertently inform researchers of the treatment they are receiving. We aim to reduce this effect by giving explicit reminders to participants before the assessment visit and by the use of self-report measures wherever feasible. Assessors record their impression of which arm of the trial each participant belongs to and their confidence in that prediction on completion of the two follow up assessments. This enables us to test whether inadvertent loss of blinding leads to bias, and to adjust for any bias that may be detected.

### Intervention

CST is an intervention for people with mild to moderate dementia, designed following extensive evaluation of the available research and is an evidence-based treatment [6]. The programme consists of fourteen 45-minute sessions which are run twice weekly. Each session incorporates use of an 'RO board', displaying both personal and orientation information, including the group name (as chosen by participants). The guiding principles of CST involve using new ideas, thoughts and associations; using orientation (but sensitively and implicitly); a focus on opinions rather than facts; using reminiscence as an aid to the here-and-now; providing triggers to aid recall; creation of continuity and consistency between sessions; focus on implicit (rather than explicit) learning; stimulating language; stimulating executive functioning and being person-centred -(treating people as unique individuals with their own personality and preferences). The CST programme aims to create an environment where people have fun, learn and where they strengthen their abilities and relationships among the group members, thus maintaining their social and cognitive skills at their optimum ability.

The Maintenance Cognitive Stimulation Therapy (CST) programme is an evidence-based maintenance group therapy programme for people with dementia. It comprises a programme of 24 sessions of maintenance CST, based on the theoretical concepts of RO/Cognitive Stimulation and grounded on the original CST programme. The programme has been developed as part of the Support at Home - Interventions to Enhance Life in Dementia (SHIELD) research programme. The aim is to create an evidence-based maintenance group therapy programme for people with dementia based on the same principles as the CST programme. A process of several different methodological approaches has been used to develop the maintenance CST programme. This includes using the preliminary findings of a feasibility study into maintenance CST [9] and data extracted from the updated Cochrane review on cognitive stimulation for people with dementia [19]. Consultation was achieved with identified experts in the field, interested academics, clinicians and service users, through holding a consensus workshop and focus groups with people with dementia, family caregivers and members of staff working directly with people with dementia [22]. A summary of the CST and maintenance CST programme can be found in Table 1.

**Table 1:** CST and maintenance CST themes.

CST Programme Session Number	Main Theme	MCST Programme Session Number
1	Physical games	8

2	Sound	7

3	My life	1 & 23

4	Food	3 & 17

5	Current affairs	2

6	Faces/Scenes	15

7	Associated Words, discussion	18

8	Being creative	4

9	Categorising objects	9

10	Orientation	19

11	Using Money (Clip Adverts II)	20

12	Number game	5

13	Word game	16 & 21

14	Team games, Quiz	6

N/A	Useful tips	11 & 24

N/A	Thinking cards	12

N/A	Art Discussion	14

N/A	Visual Clips Discussion	13

N/A	Household Treasures	10 & 22

### Recruitment and training of facilitators

Each CST and maintenance CST group has two facilitators, one from the research team and a co-facilitator who is a member of staff from the recruited centre (e.g. care home). The facilitators have at least one year of experience in dementia care. Main facilitators often have a mental health nursing, occupational therapy or clinical psychology background, experience in dementia care and group facilitation skills. The use of two facilitators for each group enables effective de-briefing and reflection to occur at the end of each session. All facilitators attend a one-day CST training developed by one of the CST pioneers (AS) as part of the dissemination strategy. The training provides a detailed background and description of CST, and uses learning methods including group observation, role-playing and small group exercise.

### Usual Care

The participants allocated to the control group receive treatment as usual. This can vary between and within centres and may change over time, but in principle, the interventions offered to this group are also available to those in the active treatment groups. Therefore, the trial examines the additional effects of maintenance CST. Our approach to costing the services and interventions received will allow us to monitor whether the treatment-as-usual group have been receiving similar therapeutic interventions. Use of antidementia medication is recorded as part of the costing information collected. It is possible that participants in the treatment-as-usual group are involved in some form of cognitive stimulation work during the 24 weeks of the study period. However, it is very unlikely that such a structured approach to CST is offered in any of the centres. It is this systematic group-based approach that is the focus of this evaluation.

#### Ethical arrangements

##### Risks and anticipated benefits for trial participants

There appear to be no documented harmful side-effects from participating in CST groups, and no serious adverse reactions were apparent in the CST study. Benefits are consistently reported by participants in the groups, including enjoyment, feelings of validation and self-worth [23]. The inclination of participants to continue meeting following the sessions provides an indication of the value placed on the benefits. Prospective participants are fully informed of the potential risks and benefits of the project.

A reporting procedure is in place to ensure that serious adverse events are reported to the Chief Investigator (MO). Upon becoming aware of an adverse event involving a participant or carer, a senior clinical member of the research team assesses its seriousness. A Serious Adverse Event (SAE) is defined in the trial as an untoward occurrence experienced by either a participant or carer which:

• results in death;

• is life threatening;

• requires hospitalisation or prolongation of existing hospitalisation;

• results in persistent or significant disability or incapacity;

• is otherwise considered medically significant by the investigator;

• falls within the scope of the Protection of Vulnerable Adults (POVA) protocol which is in place to ensure that suspected cases of abuse or neglect are followed-up in an appropriate manner.

A reporting form is submitted to the CI who assesses whether the SAE is related to the conduct of the trial and is unexpected. SAEs that are judged to be related and unexpected are reported to MREC and the trial DMEC.

### Consent

Recruited participants are in the mild to moderate stages of dementia, and would therefore generally be expected to be competent to give informed consent for participation, provided that appropriate care is taken in explaining the research and sufficient time allowed for them to reach a decision. Wherever possible a family member or other supporter is included. It is made clear to participants and family care-givers that no disadvantage will occur if they choose not to participate. In seeking consent, we follow current guidance from the British Psychological Society on evaluation of capacity. In this context, consent has to be regarded as a continuing process rather than a one-off decision, and willingness to continue participating will be continually checked through discussion with participants during the assessments. Where the participant's level of impairment increases, so that they are no longer able to provide informed consent, the provisions of the Mental Capacity Act 2005 [24] will be followed. The initial giving of informed consent can provide an indication of the person's preference for participation in the research, and the family care-giver's viewpoint can also be sought. If the person with dementia shows discomfort at any point with the assessments they can be discontinued.

### Outcome measures

Primary and secondary measures are completed at baseline (T0), after the seven weeks of the CST programme (first follow up, T1), three months after beginning of the maintenance groups (second follow-up T2) and six months after the beginning of the maintenance groups (third follow up and primary end-point T3).

#### Primary outcome measures

a) Cognition is measured using the ADAS-Cog [25]. The ADAS-Cog consists of 11 tasks measuring the disturbances of memory, language, praxis, attention and other cognitive abilities which are often referred to as the core symptoms of AD.

b) Quality of life is measured using the *Quality of Life--Alzheimer's disease Scale *[26]. The QOL-AD covers 13 domains of quality of life. It has good internal consistency, validity and reliability and its use is recommended by the European consensus on outcome measures for psychosocial interventions in dementia [27].

#### Secondary Outcomes

a) Cognition using the Mini-Mental State Examination (MMSE), [16]. The MMSE is a brief, widely used test of cognitive function, with good reliability and validity.

b) Communication is assessed using the *Holden Communication Scale *[28]. This scale is completed by staff or family caregivers and covers a range of social behaviour and communication variables.

c) Depression is assessed using the *Cornell Scale for Depression in Dementia *[29]. This scale rates depression in five broad categories using information from interviews with staff and participants. Good reliability and validity have been demonstrated.

d) Anxiety is assessed using the *Rating Anxiety in Dementia scale *[30] (RAID). This rates anxiety in four main categories and uses interviews with staff and participants. It has good validity and reliability.

e) Behaviour is assessed using the *Neuropsychiatric Inventory *(NPI) [31]. The NPI assess 10 behavioural disturbances occurring in dementia patients. Studies report that it has good validity and reliability.

f) Activities of daily living assessed using the *Alzheimer's Disease Co-operative Study - Activities of Daily Living Inventory *(ADCS-ADL) [32]. The ADCS-ADL is a structured questionnaire originally created to assess functional capacity over the range of dementia severity. The sensitivity and reliability have been establish [32].

g) Family Caregiver health is assessed using the *Short Form-12 Health Survey *(SF-12) [33]. This scale measures generic health concepts relevant across age, disease, and treatment groups. The SF-12 includes 8 concepts commonly represented in health surveys. It is a self administrative measure that provides a comprehensive, psychometrically sound, and efficient way to measure health from the patient's point of view by scoring standardized responses to standard questions. This measure is only used when a family caregiver is available from the community sample participants.

h) Costs are assessed using the validated *Client Services Receipt Inventory *(CSRI) [34], adapted for this study. Used extensively in studies of mental health and dementia [7], the CSRI gathers comprehensive data on accommodation, medication and services received. In this trial the data will cover the previous three months (at baseline and after treatment) or at three and six months follow-up. Unit costs are then attached to services and support received, based on nationally relevant estimates of long-run marginal opportunity costs. Two quality of life measures are also included for cost utility analyses. The EQ-5D [35] is a standardised instrument for use as a measure of health-related quality of life. It contains a 3-level coding system for 5 dimensions. The instrument includes a global rating of current health using a visual analog scale. The DEMQOL [36] uses self-rated reports of QoL administered by a trained interviewer; there is also a separate scale for family caregiver or members of staff reports, the DEMQOL-proxy. It includes 5 domains of quality of life. The DEMQOL has high internal consistency and acceptable inter-rater reliability and indicates concurrent validity through moderate associations with the QOL-AD and DEMQOL [36].

#### Sample Size

The primary outcome measures are cognition (ADAS-Cog) and quality of life (QoL-AD) at 24 weeks follow up. In the pilot CST trial [6] which recruited people with mild/moderate dementia (MMSE 10-24), community and institutional participants had a similar level of cognitive impairment (mean MMSE 14.5 and 14.1). The RO review [5] found a moderate effect size of 0.58 between the RO and control groups though the studies had some differences in methodology, outcome measures, and length of treatment/follow up. The MCST pilot study found a large effect size of 1.0 compared with CST alone. To detect an effect size for MCST of 0.39 on the ADASCog with power of 80% using a 5% significance level and an estimated attrition of 15% needed, a sample size of 230 at T1 is required. If an estimated 60 participants will have Alzheimer's disease and are suitable/willing to take cholinesterase inhibitors (ACHEIs), this provides sufficient numbers for the maintenance CST/ACHEIs trial platform to estimate effect size and the feasibility of the trial.

### Analyses

#### Statistical analyses of effectiveness

Analysis will be by intention to treat, in that all available data will be included. Methods of imputation such as LOCF (last observation carried forward) are of limited utility in dementia, where the expectation is decline for the usual treatment group, and participants will be lost through death and illness. Hence our sample size calculations are based on the numbers estimated to be available at the study end-point, 6 months after randomisation to either the CST only group or the maintenance CST group. Multi-level modelling will be used to address the issue of clustering within randomised groups. We shall also use analysis of covariance to adjust for baseline differences in outcome variables [37]. Analyses will consider the evaluation 6 months after the second randomisation as the primary end-point. Secondary analyses will consider the effects immediately following the CST, at 3 and 6 months. Age, gender, cholinesterase inhibitor and baseline scores on the scales being examined will be entered as covariates, together with 'centre' entered as a random factor, because treatment has been defined as participation in the group programme within the confines of the centres.

#### Economic evaluation

The primary economic evaluation is a cost-effectiveness analysis. This study also offers an opportunity to conduct a secondary cost-utility analysis. In addition, all costs and effects for people with dementia will be set out in a cost-consequence analysis. This analysis takes a public sector perspective spanning the NHS (dementia services, primary and secondary care) and social care services funded or brokered by local authorities. The interventions received will be fully costed from the perspective of local dementia services to generate a total programme cost and cost per participant. We will estimate costs from data collected in the validated Client Service Receipt Inventory (CSRI), completed with the family care-giver or care home manager. Costs will include those associated with the CST and maintenance CST groups, primary and secondary health care services used by participants in the intervention and control arms of the study (e.g. home/surgery telephone contacts with GP and practice nurse, outpatient and inpatient attendances at secondary care, prescribing), home care, residential care placements, care and support accessed through direct payments (or personal budgets), as well as indirect costs associated with caregiver time and lost productivity.

#### Incremental Cost-effectiveness Analysis

The incremental cost-effectiveness ratio (ICER) measures a ratio of costs to outcomes, with the denominator being the difference in costs between the intervention and control groups, and the numerator being the difference between these groups in an outcome measure. The primary analysis will compute the ICER for each of the primary outcomes in turn (ADAS-Cog, QOL-AD). Secondary analyses will measure outcomes using utility scores generated by the EQ5D and DEMQOL. Bootstrap calculations, will be used for examining the uncertainty in the cost-effectiveness analysis, to provide an estimate of the probability distribution of the ICER, its confidence interval, or variance in the ratio.

We will plot cost-effectiveness acceptability curves (CEACs), which have been widely adopted as a method to quantify and graphically represent uncertainty in economic evaluation studies of health care technologies. They can equally be used in the evaluation of public health interventions. Sensitivity analysis will be undertaken to test whether plausible changes in the values of the main variables affect the results of the analysis.

Cost-consequence analysis is a variant of cost-effectiveness analysis in which the components of incremental costs and consequences (health outcomes) of alternative programmes are listed without aggregation. This analysis will be used for a comparison of secondary outcome measures of participants in the intervention and control arms of the study. The inclusion of a cost-consequence analysis in addition to the standard cost-effectiveness and cost-utility analyses is to set out transparently the full range of costs and consequences resulting from CST so as to assist commissioners and policy makers responsible for funding and coordinating services.

## Discussion

This innovative and pragmatic RCT that evaluates the effectiveness and cost-effectiveness of a maintenance CST programme for dementia constitutes one of the largest and longest trials for people with dementia and one of the first including an integrated cost-effective analysis of a psychosocial intervention. This study reflects the current emphasis on improving cognition and quality of life using psychosocial interventions, and the emphasis is on working under a person-centred framework through group exercises aiming at global stimulation of cognitive abilities for a prolonged period of time. The NICE-SCIE guidelines [8] on the management of dementia offer few evidence-based recommendations about psychosocial approaches. This is mainly due to the lack of high-quality studies showing their efficacy and lack of specific guidance for the duration that psychosocial interventions should be provided. The results of this trial may therefore contribute to future practice guidelines and, if successful, will help the widespread use of psychosocial interventions in dementia care such as CST within health and social care services [38]. CST has now been evaluated in a number of countries [21,39,40] and the UK National Audit Office report highlighted that CST was available in 29% of community mental health teams for older people [41].

This study should provide definitive evidence of the potential efficacy of maintenance CST and establish how far the long-term efficacy can be compared with antidementia drugs such as cholinesterase inhibitors in terms of cognition and quality of life. The results of this study, both in terms of efficacy and cost-effectiveness, are likely to influence the availability and provision of maintenance CST in the UK and internationally, and will also impact on evidence-based guidelines and strategic policies in dementia care.

## Competing interests

AS runs the CST training course on a commercial basis.

AS, BW and MO have co-authored a CST manual, the royalties from which are received by the Dementia Services Development Centre Wales.

## Authors' contributions

MO, RTW, ITR developed the original concept of the trial, and EA and MO drafted the original protocol; ITR developed the design and methodology; MK developed the health economic component; AS and JH co authored the treatment manual; all authors reviewed and commented on drafts of the protocol and paper.

## Appendix 1

### Inclusion and exclusion criteria

#### Inclusion criteria

All participants are people with dementia who:

◦ meet the DSM-IV criteria for dementia of any type, including Alzheimer's, vascular, Lewy Body type and mixed

◦ are in the mild to moderate stage of dementia (Clinical Dementia Rating)

◦ can communicate and understand communication in English

◦ can engage in group activity for at least 45 minutes

#### Exclusion criteria

Participants do not have any characteristic which could affect participation, e.g.: major physical illness; sensory impairment; disability or high level of agitation.
